# Development and Preliminary Evaluation of an AI Chatbot-Based Psychosocial Self-Management Program for Women with Infertility: A Non-Randomized Feasibility Study

**DOI:** 10.3390/bs16101956

**Published:** 2026-10-21

**Authors:** Jungmin Lee, Seurk Park, Soo-Hyun Nam

**Affiliations:** 1 School of Nursing, Hallym University, Chuncheon 24252, Gangwon-do, Republic of Korea; j_lee0624@hallym.ac.kr; 2 Global Frontier Research Center, Hallym University, Chuncheon 24252, Gangwon-do, Republic of Korea; 3 School of Nursing Science, Gyeongkuk National University, 1375 Gyeongdong-ro, Andong 36729, Gyeongsangbuk-do, Republic of Korea

**Keywords:** infertility, artificial intelligence, chatbot, psychosocial intervention, self-management, alexithymia, feasibility study, women’s health

## Abstract

Background: Women undergoing infertility treatment experience elevated perceived stress, anxiety, and difficulty identifying and describing their emotions (alexithymia), yet access to conventional psychological care is constrained by stigma, time, and cost. Objective: To develop an artificial intelligence (AI) chatbot-based psychosocial self-management program for women with infertility and to examine its feasibility and preliminary patterns of change in perceived stress, depressive symptoms, state anxiety, and alexithymia. Feasibility was the primary objective; the four psychological measures were secondary and are reported as exploratory. Methods: A non-randomized, two-group pretest–posttest feasibility study was conducted with 37 women recruited sequentially from a single infertility centre in the Republic of Korea; the comparison group was recruited and assessed first (*n* = 19), followed by the intervention group (*n* = 18). The intervention group completed six chatbot sessions over two weeks. Outcomes were the Perceived Stress Scale (PSS), Patient Health Questionnaire-9 (PHQ-9), State-Trait Anxiety Inventory–State subscale (STAI-S), and the 20-item Toronto Alexithymia Scale (TAS-20). Results: Feasibility indicators were favourable: retention was 92.5% overall (37/40) and 90.0% in the intervention group (18/20), and all retained intervention participants completed all six sessions (108/108, 100%). The groups differed at baseline in age (33.33 ± 3.36 vs. 35.84 ± 4.00 years, *p* = 0.047). Group × time interactions favoured the intervention group for perceived stress (F(1, 35) = 14.37, *p* < 0.001, η_p_^2^ = 0.29), state anxiety (F(1, 35) = 4.40, *p* = 0.043, η_p_^2^ = 0.11), and alexithymia (F(1, 35) = 6.92, *p* = 0.013, η_p_^2^ = 0.16), but not for depressive symptoms (F(1, 35) = 3.66, *p* = 0.064, η_p_^2^ = 0.09). Conclusions: The program was feasible in terms of retention and adherence, with all retained intervention participants completing all six sessions. Because allocation was non-random and confounded with calendar time, and because the sample was small, these findings describe preliminary patterns of change and do not establish effectiveness. An adequately powered randomized trial with concurrent recruitment, an attention-matched comparator, and follow-up assessment is required.

## 1. Introduction

Infertility is defined as the inability of a couple of reproductive age to conceive after one year of regular, unprotected intercourse (Zegers-Hochschild et al., 2017) and has been identified by the World Health Organization (WHO) as a global public health issue (World Health Organization, 2018). According to Statistics Korea, the total fertility rate in the Republic of Korea fell from 1.23 to 0.72 in 2023, the lowest among OECD member countries (Korean Statistical Information Service, 2024). As the age at marriage and childbirth continues to rise, more women are experiencing infertility. To address this issue, the Korean government has implemented infertility treatment support programs, and both financial and insurance coverage have expanded (M. O. Kim et al., 2019). Nonetheless, the number of individuals diagnosed with infertility continues to grow by more than 2.2% annually (Health Insurance Review and Assessment Service, 2022).

Women with infertility often face multifaceted challenges, including despair over not conceiving, guilt from not achieving developmental milestones, and shame related to sexual activity during treatment (J. Lee et al., 2025; M. K. Kim et al., 2014). Physical pain and financial burdens from diagnosis and prolonged treatment further intensify distress (Khalesi & Kenarsari, 2024). The unpredictability and lack of control inherent in infertility frequently lead to severe emotional distress, contributing to chronic anxiety and depression (Ha et al., 2020; M. K. Kim et al., 2014). Even after conception through assisted reproductive technologies, miscarriage risk and other uncertainties can heighten psychological instability (Verhaak et al., 2001). Previous studies show that 94.6% of women undergoing infertility treatment report depressive symptoms (Hwang, 2011), and their psychological distress is comparable to that of individuals with cardiovascular disease or cancer (Gameiro et al., 2012).

Infertility-related stress not only reduces the success of infertility treatment but also interferes with maintaining a healthy pregnancy after conception (Frederiksen et al., 2015). Psychosocial intervention and emotional support are therefore critical for treatment success and maternal health (Ha et al., 2022). Psychological interventions such as cognitive behavioural therapy, mind–body practices, and art therapy have been shown to reduce distress, improve quality of life, and increase conception rates in women with infertility (Frederiksen et al., 2015; Gaitzsch et al., 2020; LoGiudice & Massaro, 2018). A meta-analysis confirmed that reducing anxiety and depression in this population significantly improved intrauterine insemination outcomes and pregnancy rates (Ha et al., 2022).

However, existing psychological support programs for women with infertility face limitations, including provider fatigue, time and financial constraints, stigma, fear, and participants’ emotional challenges. High dependence on human facilitators also restricts participants’ autonomy in managing their mental health (Lucas et al., 2014). Chatbot-based interventions have been proposed as one response to these constraints (Greer et al., 2019; A. R. Lee et al., 2019; Y. C. Lee et al., 2020). Chatbots offer immediate, interactive conversation and continuous availability (Y. C. Lee et al., 2020); they promote self-disclosure and reduce psychological conflict (Y. C. Lee et al., 2020); and, unlike face-to-face therapy, they provide anonymity, reduce concerns about negative evaluation, and enable freer expression of sensitive emotions (A. R. Lee et al., 2019).

More recent evidence allows this rationale to be stated with greater precision. A PRISMA-compliant systematic review of 35 studies on AI-enabled tools for self-directed stress management identified five recurring user-facing functions—psychological intervention, behavioural support, psychoeducation, emotional companionship, and stress monitoring and triage—and concluded that such systems show preliminary promise while evidence for their longer-term effectiveness remains limited (Reyes et al., 2026). That review is directly relevant to the present work in two respects. First, it provides an empirical basis for selecting which functions a chatbot for this population should perform, rather than relying on general claims about conversational agents. Second, it makes explicit that the current state of the field supports feasibility and short-term signal detection rather than established efficacy, and that studies in this area are frequently limited by small samples, non-randomized designs, and the absence of follow-up. The present study was designed and is reported with that distinction in mind.

In the Republic of Korea, although research on psychosocial interventions for women with infertility has increased since 2011 (Hwang, 2011), few studies have applied AI-based chatbot interventions specifically to mental health self-management in this population. The aims of the present study were therefore (a) to develop an interactive AI chatbot-based psychosocial self-management program grounded in the functions identified above and tailored to infertility-related stigma, and (b) to examine its feasibility and the preliminary pattern of change in perceived stress, depressive symptoms, state anxiety, and alexithymia. Consistent with the design described below, the study was not intended to establish effectiveness. The two aims are not of equal standing. Feasibility—whether women in active fertility treatment would enrol in, remain in, and complete a six-session generative-AI program, and whether it could be delivered safely—was the primary objective. The four psychological measures were secondary, and because no hierarchy among them was specified in advance, all four are reported as exploratory.

## 2. Methods

### 2.1. Design

This was a non-randomized, two-group pretest–posttest feasibility study. Participants were allocated to the intervention or comparison condition by recruitment period rather than by randomization: the comparison group was recruited and assessed first, and the intervention group was recruited subsequently. This sequential approach was adopted because both groups attended a single infertility centre, and concurrent allocation would have created a substantial risk of contamination between participants who did and did not receive the chatbot.

The comparison condition consisted of usual clinic care with no study activity. No attention-matched or sham condition was provided, so the two conditions differed not only in the chatbot content but also in contact frequency, scheduled attention, expectancy, and exposure to a novel technology. Any difference observed between them is therefore attributable to the intervention package as a whole rather than to its active psychological components.

The objective hierarchy was specified accordingly. The primary outcomes were the feasibility indicators defined in Section 2.4.4, including recruitment, retention, adherence and session completion, engagement, and adverse events. The four psychological measures were designated as secondary exploratory outcomes, and no primary psychological endpoint was prespecified before data collection. Accordingly, these psychological outcomes were interpreted as preliminary estimates of potential intervention-related changes rather than as confirmatory efficacy outcomes. Effect sizes and 95% confidence intervals were reported alongside *p*-values to characterize the magnitude and precision of the observed between-group differences in change.

### 2.2. Participants

Participants were women receiving care at a single infertility centre in the Republic of Korea. Eligibility was limited to those who fully understood the study purpose, voluntarily agreed to participate, and provided written informed consent.

#### Eligibility Criteria

Inclusion criteria were: (1) women with a clinician-confirmed diagnosis of infertility made in routine care at the participating centre; and (2) women who had undergone at least one infertility-related medical procedure (e.g., ovulation induction, hormone replacement therapy, intrauterine insemination, or in vitro fertilization) within the past 12 months. Exclusion criteria were: (1) receipt of psychological counselling or psychiatric treatment for infertility-related stress within the past 12 months; and (2) current use of antidepressants.

In the IRB-approved protocol, infertility was defined according to the general definition of failure to conceive after at least 12 months of regular unprotected intercourse. After recruitment began, this criterion was operationalized in more specific terms according to the participating centre’s routine diagnostic practice, under which the 12-month threshold is applied to women younger than 35 years, a six-month threshold is applied to women aged 35 years or older, and a diagnosis may be made earlier where a contributing factor has been identified. Eligibility was therefore confirmed on the basis of the clinician’s diagnosis made in routine care at the centre rather than by applying a uniform 12-month threshold.

Sample size was estimated a priori from a previous psychological nursing intervention for women with infertility that reported d = 0.96, converted to f = 0.67 for repeated-measures analysis of variance. With G*Power 3.1.9, α = 0.05, and power (1 − β) = 0.80, the minimum requirement was 19 per group (total *N* = 38). Twenty participants were enrolled in each group; one comparison participant did not complete the posttest and two intervention participants withdrew consent after the pretest, yielding 19 and 18 participants, respectively. The final intervention group of 18 falls one participant short of the a priori requirement.

### 2.3. Instruments

#### 2.3.1. Perceived Stress—Perceived Stress Scale (PSS)

Perceived stress was measured with the PSS (Cohen et al., 1983). This 10-item scale assesses the extent to which life situations are appraised as unpredictable, uncontrollable, and overloading. Items are rated from 0 (“never”) to 4 (“very often”) for the past month; items 4, 5, 7, and 8 are reverse-scored. Total scores range from 0 to 40, with higher scores indicating greater perceived stress. Cronbach’s α was 0.86 in the original study and 0.91 in the present study.

#### 2.3.2. Depressive Symptoms—Patient Health Questionnaire-9 (PHQ-9)

Depressive symptoms were measured with the Korean version of the PHQ-9 (Kroenke & Spitzer, 2002). Each of the nine items is rated from 0 (“not at all”) to 3 (“nearly every day”) for the past two weeks. Total scores range from 0 to 27, with higher scores indicating more severe symptoms. Cronbach’s α was 0.89 in the original study and 0.90 in the present study.

#### 2.3.3. State Anxiety—State-Trait Anxiety Inventory, State Subscale (STAI-S)

State anxiety was assessed with the STAI-S (Spielberger et al., 1970). This 20-item scale measures anxiety experienced at the present moment. Items are rated from 1 (“not at all”) to 4 (“very much so”); items 1, 2, 5, 8, 10, 11, 15, 16, 19, and 20 are reverse-scored. Total scores range from 20 to 80, with higher scores indicating greater state anxiety. Cronbach’s α was 0.91 in the original study and 0.92 in the present study.

#### 2.3.4. Alexithymia—20-Item Toronto Alexithymia Scale (TAS-20)

The TAS-20 (Bagby et al., 1994) was administered. The TAS-20 is a measure of alexithymia and comprises three facets: difficulty identifying feelings, difficulty describing feelings, and externally oriented thinking. It indexes the capacity to identify and verbalize emotional experience; it is not a direct behavioural measure of emotional expression or disclosure. Throughout the present version the construct is reported as alexithymia, or as difficulty identifying and describing emotions, and a decrease in TAS-20 scores is interpreted as a reduction in alexithymia rather than as an increase in expressive behaviour. Items are rated from 1 (“strongly disagree”) to 5 (“strongly agree”); items 4, 5, 10, 18, and 19 are reverse-scored. Total scores range from 20 to 100, with higher scores indicating greater alexithymia. Although originally developed in psychosomatic samples, the TAS-20 has been widely applied in non-clinical populations (Sifneos, 1988) and is independent of clinical diagnosis. Cronbach’s α was 0.81 in the original study and 0.87 in the present study.

### 2.4. The Chatbot-Based Psychosocial Self-Management Program

#### 2.4.1. Development and Content Validation

The program was developed in the following stages. An initial version of the session structure, the empathic interaction templates, and the system prompt was drafted by the research team on the basis of the design principles set out below. The draft was then reviewed by three experts—a women’s health nursing professor, a professor of psychiatric and mental health nursing, and a mental health nurse specialist—who assessed each component for clinical appropriateness, stigma sensitivity, and fidelity to the intended therapeutic strategy. Items rated as unclear or potentially stigmatizing were revised, and the system prompt was amended accordingly.

The revised program was pilot tested with three women meeting the eligibility criteria who did not take part in the main study. Pilot testing examined comprehensibility of the chatbot’s language, the pacing and length of sessions, and technical operation.

#### 2.4.2. Design of the Intervention Content

The chatbot’s functions were organized around the domains identified in reviews of AI chatbot-based digital mental health interventions—screening and triage, content delivery, and symptom management (Boucher et al., 2021)—and were mapped to the five functions synthesized by Reyes et al. (2026): psychological intervention, behavioural support, psychoeducation, emotional companionship, and stress monitoring and triage. Before these functions were applied, the program was oriented to the inner emotional world and stigma-related experiences of women with infertility. Non-stigmatizing language (e.g., “trying to conceive” rather than “infertility”) was used, and participants were encouraged to express emotions openly. An emotional-processing strategy was applied in three stages: identifying emotions; providing empathy and validation; and offering a psychoeducational explanation linking thoughts, emotions, and behaviours.

The screening function was not intended for medical diagnosis but for identifying distress through structured questions so that tailored feedback could be given (Balaskas et al., 2021). The symptom-management function supported recognition of symptom and behaviour patterns (e.g., inactivity, sleep disturbance, anxiety, depressed mood, stress). For each target emotion (depressed mood, anxiety, stress, anger, and loneliness), three to four empathic interaction templates were specified. Practical emotion-regulation resources were provided, including a 10-min mindfulness meditation, a paced-breathing exercise, guided imagery, and cognitive restructuring prompts. Participants completed six sessions over two weeks (three per week); an automated scheduling prompt directed users to the next session.

#### 2.4.3. Technical Specification

The program was implemented as a purpose-built conversational application, created by the research team as a Claude-powered artifact on the Claude platform (claude.ai; Anthropic, PBC, San Francisco, CA, USA). Participants did not use the general Claude chat interface. The underlying model was Claude Sonnet 4. Participants accessed the application through a shared link provided by the research team and signed in with their own Claude account. The application displayed only the study chatbot; no model selection, web search, file upload, or other general-purpose features were available to participants. Neither the application code nor the system prompt was changed during the data-collection period. Conversational history was retained within a session and was not carried across sessions. The chatbot was delivered in Korean.

The system prompt specified the chatbot’s role, the stigma-sensitive language rules, the three-stage emotional-processing sequence, the session structure, and explicit safety constraints: the model was instructed not to provide medical, pharmacological, or prognostic advice; not to comment on any participant’s likelihood of conception; and to direct the participant to the research nurse and to national crisis services if suicidal ideation or acute distress was expressed.

#### 2.4.4. Feasibility Measures

Feasibility was the primary objective and was operationalized across five domains, following conventional reporting for feasibility studies: (i) recruitment—number of women who responded to the recruitment notice and were screened, number enrolled, and the recruitment window; (ii) retention—the proportion of enrolled participants completing the posttest, by group; (iii) adherence—the proportion of prescribed sessions completed, and the proportion of participants completing all six, both based on participant self-report; (iv) engagement—self-reported session duration; (v) adverse events—any participant-reported safety incident, including model responses judged inappropriate, and any escalation under the procedure described in Section 2.4.5.

#### 2.4.5. Safety Procedures

Because the program was designed to facilitate the expression of psychological distress and respond to such expressions, the following safeguards were established before data collection. Response generation: All conversational responses were generated by the model at runtime. The system prompt, stigma-sensitive language rules, and empathic interaction templates constrained the content and sequence of each session; however, the responses were not drawn from a predetermined fixed script.

The system prompt instructed the model to acknowledge expressions of suicidal ideation, self-harm, or acute distress, provide information about the national crisis counselling hotline, and recommend that participants contact the research nurse identified at enrolment. No participant-reported harm or safety incidents occurred during the study period.

#### 2.4.6. Data Governance and Privacy

The use of Anthropic’s Claude model was specified in the approved protocol and participants were informed at enrolment that the program was built on Claude, a generative AI model developed by Anthropic. Because the application ran on a commercial large language model platform, conversational content entered by participants was transmitted to and processed by the model provider (Anthropic). Each participant accessed the application through their own Claude account, and provider-side data retention and use were governed by the provider’s terms of service and privacy policy, to which participants agreed when creating or using their account. Because conversations were processed within participants’ own accounts, the research team did not collect, export, or store any conversation content, and study data were limited to questionnaire responses and self-reported session records. To minimise the disclosure of identifiable information, participants were instructed at enrolment not to enter their name, contact details, hospital or clinic name, or other identifying information. However, neither the approved protocol nor the participant information sheet contained a separate, explicit statement on provider-side data processing, retention, or potential use for model training.

### 2.5. Data Collection

The study was approved by the Institutional Review Board of K University (Approval No. 1040191-202504-HR-009-01; approved 30 April 2025). Given the stigma surrounding infertility, participants were fully informed about the definition of infertility and the nature of the intervention, and care was taken to avoid reinforcing negative perceptions. The consent form covered the study purpose, intervention content, group assignment, data use, confidentiality, and participant rights. Participation was voluntary and could be withdrawn at any time without penalty.

Participants were recruited from a single infertility centre in K province between June and August 2025 in response to recruitment notices. Recruitment proceeded in two consecutive blocks. The comparison group was recruited and completed the pretest first (June to July 2025), followed two weeks later by the posttest without any study intervention; participants in this group continued to receive usual clinic care. The intervention group was then recruited (July to August 2025), completed the pretest, received the six-session chatbot program over two weeks, and completed the posttest immediately after the final session. No participant contributed to both conditions. Because the two blocks were assessed at different calendar periods, any secular change at the clinic, in the participant mix, or in treatment-cycle timing during the study window is indistinguishable from an intervention effect. Trained research assistants conducted data collection and coding; each assessment took approximately 10–15 min. All participants received refreshments and a small gift.

Comparison participants completed the posttest online, two weeks after the pretest and with no study contact in between. Intervention participants completed the posttest online, immediately after their final chatbot session.

### 2.6. Data Analysis

Data were analysed using IBM SPSS Statistics version 29.0. Normality was assessed using the Kolmogorov–Smirnov test. Baseline characteristics were compared using chi-square tests, Fisher’s exact tests, and independent-samples *t*-tests, as appropriate. Changes in outcome measures from pretest to posttest were examined using two-way repeated-measures analysis of variance (ANOVA), with group, time, and the group × time interaction included in the model. Effect sizes were reported using partial eta squared (ηp^2^) for ANOVA effects and Cohen’s d for between-group differences in change scores. Statistical significance was set at *p* < 0.05.

Because the study groups were not randomized and differed numerically in prior failed assisted reproductive technology (ART) cycles, analysis of covariance (ANCOVA) was additionally conducted for each outcome, with the posttest score as the dependent variable, group as the between-subjects factor, and the corresponding pretest score and prior failed ART experience as covariates. Two additional sensitivity analyses were performed: the ANCOVA models were re-estimated with age added as a covariate, and the analysis was repeated in participants reporting an infertility duration of one year or more.

## 3. Results

### 3.1. Baseline Sociodemographic Characteristics

The groups were not equivalent at baseline. Mean age was 33.33 ± 3.36 years in the intervention group and 35.84 ± 4.00 years in the comparison group, a statistically significant difference (t = −2.06, *p* = 0.047). Prior failed ART cycles were numerically more frequent in the comparison group (31.6%) than in the intervention group (5.6%), although this difference did not reach statistical significance (Fisher’s exact *p* = 0.090). No other sociodemographic variable differed significantly between groups: occupation (88.9% and 89.5% employed, *p* = 1.000), religious affiliation (*p* = 0.447), economic status (*p* = 1.000), self-reported infertility duration (*p* = 0.773), and having children (44.4% and 57.9%, *p* = 0.413) (Table 1).

### 3.2. Baseline Comparability of Outcome Variables

All outcome variables were normally distributed by the Kolmogorov–Smirnov test. Independent *t*-tests showed no statistically significant baseline differences between groups in perceived stress (t = −0.82, *p* = 0.42), depressive symptoms (t = −0.79, *p* = 0.43), state anxiety (t = −1.29, *p* = 0.21), or alexithymia (t = −0.44, *p* = 0.66); in every case the comparison group scored numerically higher at baseline (Table 2). As noted in Section 3.1, non-significance here should not be read as evidence of equivalence.

### 3.3. Feasibility Outcomes

Feasibility outcomes are summarized in Table 3. Participants were recruited through a recruitment notice at a single fertility centre from June to August 2025. Forty women who responded to the notice during this period were screened; all met the eligibility criteria and consented to participate, and none were excluded or declined. All 40 were enrolled (20 per condition). Because the number of women who saw the notice was not recorded, a recruitment rate relative to the eligible population could not be calculated. Retention was 92.5% overall (37/40): 19 of 20 comparison participants completed the posttest, and 18 of 20 intervention participants did so, two having withdrawn consent after the pretest. Based on participant self-report, all 18 retained intervention participants completed all six sessions (108 of 108 prescribed sessions; 100%) (Table 3).

### 3.4. Descriptive Change, Between-Group Differences, and Effect Sizes

Table 4 presents the pretest and posttest descriptive statistics, within-group mean changes, between-group differences in change with their 95% confidence intervals, and Cohen’s d.

First, the between-group differences in change were −5.91 points for perceived stress (95% CI, −9.07 to −2.74; d = −1.25), −7.05 points for alexithymia (95% CI, −12.50 to −1.61; d = −0.86), and −3.81 points for state anxiety (95% CI, −7.49 to −0.12; d = −0.69), corresponding to large effect sizes for perceived stress and alexithymia and a moderate effect size for state anxiety.

Second, depressive symptoms showed a different pattern. The comparison group showed a mean decrease of 2.16 points (paired t = −3.19, *p* = 0.005), whereas the intervention group showed no significant change (−0.28 points, *p* = 0.70). The between-group difference in change was +1.88 points, favouring the comparison group (95% CI, −0.11 to 3.87; d = 0.63).

Third, for perceived stress, state anxiety, and alexithymia, the comparison group’s scores increased by 1.63, 1.47, and 2.05 points, respectively, whereas the intervention group’s scores decreased by 4.28, 2.33, and 5.00 points, respectively.

### 3.5. Repeated-Measures Analysis of Variance

Table 5 presents the results of the two-way repeated-measures ANOVA. Group × time interactions were statistically significant for perceived stress, F(1, 35) = 14.37, *p* < 0.001, ηp^2^ = 0.29; state anxiety, F(1, 35) = 4.40, *p* = 0.043, ηp^2^ = 0.11; and alexithymia, F(1, 35) = 6.92, *p* = 0.013, ηp^2^ = 0.16. The interaction for depressive symptoms was not significant, F(1, 35) = 3.66, *p* = 0.064, ηp^2^ = 0.09, and its direction favoured the comparison group rather than the intervention group. Significant between-subjects group effects were present for perceived stress, F(1, 35) = 10.41, *p* = 0.003, ηp^2^ = 0.23, and state anxiety, F(1, 35) = 4.86, *p* = 0.034, ηp^2^ = 0.12, indicating that the comparison group scored higher on average across both assessments.

### 3.6. Sensitivity Analyses

Analysis of covariance was conducted for each outcome with the posttest score as the dependent variable and group, the corresponding pretest score, and prior failed ART experience as predictors (Table 6). Adjustment did not change the pattern obtained in the primary analyses. The adjusted difference favoured the intervention group for perceived stress (−6.69; 95% CI −9.64 to −3.74; *p* < 0.001), alexithymia (−6.85; −12.07 to −1.64; *p* = 0.012) and state anxiety (−4.21; −8.23 to −0.20; *p* = 0.040), and was small, non-significant and in the opposite direction for depressive symptoms (+0.82; −0.74 to +2.38; *p* = 0.292). Prior failed ART was itself associated with lower posttest depressive symptoms (b = −1.99, *p* = 0.048) but not with the other outcomes.

Because the groups also differed in age (Section 3.1), the models were re-estimated with age added as a covariate. The results were essentially unchanged: adjusted differences were −7.02 for perceived stress (95% CI −10.13 to −3.91; *p* < 0.001), −6.84 for alexithymia (−12.37 to −1.32; *p* = 0.017), −4.56 for state anxiety (−8.72 to −0.41; *p* = 0.032), and +0.87 for depressive symptoms (−0.79 to 2.52; *p* = 0.294).

An exploratory analysis restricted to the 12 participants reporting an infertility duration of one year or more (4 intervention, 8 comparison) showed that the difference in change remained significant for perceived stress (−9.87, *p* = 0.015) but not for the other three outcomes. Given the very small subgroup, this finding should be interpreted with caution.

## 4. Discussion

### 4.1. Summary and Interpretation

The primary finding of this study concerns the feasibility of delivering the intervention to women undergoing active fertility treatment. Forty participants were enrolled over the three-month recruitment window, 90% of those allocated to the intervention remained in the study, and all retained participants completed all six sessions without a missed session or safety escalation. Considering the sustained psychological and logistical burden associated with infertility treatment, as well as the relative novelty of the chatbot-based delivery format for many participants, this level of adherence provides important preliminary evidence that the intervention can be delivered and completed as intended. This finding provides a practical basis for proceeding to a larger controlled trial. However, feasibility should be interpreted cautiously because adherence alone does not fully capture participants’ acceptability or perceived usability of the intervention. In the absence of validated measures of acceptability and usability, completion of all sessions indicates that participants were able to engage with and tolerate the intervention but does not establish the extent to which they perceived it as useful, satisfactory, or acceptable. Future studies should therefore incorporate validated acceptability and usability measures alongside objective engagement indicators to provide a more comprehensive assessment of intervention feasibility.

This study developed an AI chatbot-based psychosocial self-management program for women with infertility and examined its feasibility together with preliminary patterns of change. The program was fully completed by all retained intervention participants, and group × time interactions favoured the intervention condition for perceived stress, state anxiety, and alexithymia, with a non-significant interaction in the opposite direction, favouring the comparison group, for depressive symptoms. The following sections discuss these findings in relation to depressive symptoms, perceived stress, state anxiety, and alexithymia, respectively.

### 4.2. Depressive Symptoms

Depressive symptoms did not follow the pattern of the other outcomes. The comparison group improved by 2.16 points over the two weeks (*p* = 0.005) while the intervention group changed by −0.28 points (*p* = 0.70), so the non-significant interaction (*p* = 0.064) favoured the comparison group, and covariate adjustment left a small non-significant difference in the same direction (+0.82; 95% CI −0.74 to +2.38).

The absence of a significant between-group difference in depressive symptoms may be interpreted from several perspectives. First, the relatively brief and low-intensity intervention used in the present study may have been insufficient to produce meaningful changes in depressive symptoms. Previous meta-analyses have suggested that improving depressive symptoms among women experiencing infertility may require more structured, intensive, or longer-term interventions, such as cognitive behavioural therapy or mind–body relaxation approaches (Chan et al., 2006; Frederiksen et al., 2015). From this perspective, a chatbot-based intervention may have limitations in replicating the depth and duration of structured psychotherapy. Second, the improvement observed in the comparison group may have partly reflected regression to the mean, given their relatively higher baseline depressive symptom scores, or temporal factors that influenced participants at a particular point during the study period. However, because participants were not randomly allocated and the two groups were recruited and assessed at different time points, these possibilities cannot be clearly distinguished in the present study. Third, the sample size may have been insufficient to detect the observed effect with adequate statistical power. Therefore, the present findings should not be interpreted as evidence that the intervention is ineffective for depressive symptoms; rather, they suggest that the evidence was insufficient to clearly determine its effect on depressive symptoms given the brief intervention period, non-randomized design, and limited sample size. Notably, the significant group × time interactions observed for perceived stress, state anxiety, and alexithymia, but not for depressive symptoms, suggest that the effects of a brief generative AI-based intervention may not occur uniformly across psychological outcomes but may vary according to the target symptom, intervention intensity, participants’ baseline characteristics, and study design.

### 4.3. Stress, State Anxiety, and Alexithymia

The larger differential changes in perceived stress and alexithymia may reflect the suitability of the program for immediate and situational emotional burden. Women experiencing infertility report substantial psychosocial distress exacerbated by internalized and social stigma (J. Lee et al., 2025; Ying et al., 2015; Zurlo et al., 2018). The chatbot offered an anonymous and non-judgemental space for emotional disclosure, and prior work indicates that digital interventions offering anonymity can support emotional expression and psychological safety (Fitzpatrick et al., 2017). The combination of empathic messaging with mindfulness, paced breathing, and cognitive restructuring may have facilitated emotional processing, and immediate availability at moments of distress may have contributed further.

Regarding state anxiety, the group × time interaction was statistically significant (*p* = 0.043; ANCOVA-adjusted *p* = 0.040). State anxiety is by definition a transient condition that fluctuates with circumstances, in contrast to trait anxiety (Spielberger et al., 1970). Brief techniques such as mindfulness, Tai Chi, and paced breathing have produced short-term reductions in acute anxiety (Chen et al., 2021; Akdağ et al., 2025): a single 30-min Tai Chi session reduced anxiety in nursing students (Chen et al., 2021), and a 12-min slow-breathing session produced immediate reductions in STAI-S scores in adolescents (Akdağ et al., 2025). Because the posttest in the present study was administered immediately after the final session, the observed change may partly reflect an acute, session-proximal state rather than a durable reduction in anxiety. Future studies should incorporate follow-up assessments at multiple time points after the intervention to determine whether the observed reduction in anxiety is sustained over time. Longitudinal follow-up assessments would therefore help distinguish immediate intervention effects from sustained changes and provide a clearer understanding of the durability of the intervention effect.

Taken together, the favourable group × time interactions for perceived stress, state anxiety, and alexithymia observed in the present study suggest that the interactive chatbot format may be a promising means of delivering brief psychosocial support to women experiencing infertility. However, whether the addition of generative AI itself provides benefits beyond the underlying psychological techniques remains an important empirical question. Evidence from previous studies is mixed. Hung et al. (2025) found that adding an AI chatbot to brief gratitude and self-affirmation activities did not improve the primary well-being outcomes compared with equivalent non-AI activities, although benefits were observed for one exploratory outcome. Reyes et al. (2026) similarly reported preliminary potential but limited evidence regarding the longer-term effectiveness of generative AI-based psychological interventions. These findings do not diminish the changes observed in the present study; rather, they suggest that the value of generative AI may depend on how its interactive features complement the therapeutic content. In the context of infertility, where emotional experiences may be highly personal and difficult to disclose, the conversational format may have provided an additional channel through which participants could engage with and elaborate on the intervention content. Nevertheless, because the present study did not include a non-AI version of the same intervention or an attention-matched comparison condition, the relative contributions of the intervention content, engagement with the intervention, and the conversational AI component cannot be disentangled. Future controlled studies should therefore compare the AI chatbot with a content- and attention-matched non-AI self-help condition to determine whether the interactive component provides incremental benefits and to identify the circumstances in which generative AI may meaningfully augment brief psychosocial interventions.

### 4.4. Limitations

First, allocation was not randomized and was confounded with recruitment period. The comparison group was recruited and assessed before the intervention group, so any change over the study window is indistinguishable from an intervention effect. Accordingly, this study describes preliminary patterns of change.

Second, the study was underpowered relative to its own plan. The a priori requirement of 19 participants per group was not met in the intervention group (*n* = 18), and the recruitment target of 40 participants was itself inadequate to accommodate 15% attrition from the required sample of 38.

Third, the feasibility assessment was incomplete. Acceptability and usability were not measured using validated instruments. Because the number of women who saw the recruitment notice was not recorded, a recruitment rate relative to the eligible population could not be calculated, although all women who responded were eligible and enrolled. In addition, because conversations were processed within participants’ own accounts and were not accessible to the research team, adherence and session duration were based on self-report, and the appropriateness of model responses could not be audited directly; these indicators may therefore be subject to recall or social desirability bias.

Fourth, outcome timing and construct coverage were limited. The posttest was administered immediately after the final session, so session-proximal state effects cannot be separated from durable change, and no follow-up assessment was conducted. Outcomes were self-reported, and participants in the intervention group were unblinded and aware that they were receiving a novel program, so demand characteristics may have inflated reported improvement. The TAS-20 indexes alexithymia rather than expressive behaviour; therefore, the present findings reflect self-reported capacity to identify and describe emotions rather than observed emotional disclosure.

Fifth, the sample may limit generalisability. Participants were recruited through a notice at a single fertility centre, and women who volunteered may have been more motivated or more open to a chatbot-based intervention than the wider population of women with infertility.

Finally, although participants were informed that the program ran on Anthropic’s Claude model and used it under their own accounts and the provider’s terms, the consent documents did not separately and explicitly describe the processing of conversational content by a third-party model provider, including provider-side retention and possible use for model training. Future studies using commercial large language model platforms should state these elements explicitly in the consent process.

### 4.5. Implications

Within these constraints, the study makes several contributions. Practically, it demonstrates that a six-session generative-AI program can be delivered to women in active fertility treatment with complete session adherence and without safety events, which is a precondition for any larger trial. Clinically, it addresses a documented gap for women who are unable or unwilling to voice emotional distress because of stigma, by offering a private, non-judgemental space that may complement rather than replace professional care. The pattern of results also indicates where such a program is and is not likely to help: the signal was strongest for perceived stress and alexithymia, weaker for state anxiety, and absent for depressive symptoms.

## 5. Conclusions

An AI chatbot-based psychosocial self-management program for women with infertility was developed and delivered with complete session adherence among retained participants in a small, non-randomized sample. Differential change favoured the intervention condition for perceived stress, state anxiety, and alexithymia; for depressive symptoms, the comparison group improved and the intervention group did not. Because allocation was determined by recruitment period rather than randomization, because part of the divergence reflects deterioration in the comparison group, because the groups were not equivalent at baseline, and because the sample was small and assessed only immediately after the intervention, these results constitute preliminary evidence of feasibility (retention and adherence) and preliminary patterns of change—not evidence of effectiveness. The findings support proceeding to a randomized controlled trial with concurrent recruitment, an attention- and content-matched comparator, identical assessment procedures in both arms, an adequate sample, and follow-up assessment; such a trial should also help identify the conditions under which a conversational AI component is most likely to add value beyond the underlying psychological technique.

## Figures and Tables

**Table 1:** Baseline sociodemographic characteristics of the intervention and comparison groups.

Variables	Categories	Intervention (*n* = 18)	Comparison (*n* = 19)	Total (*N* = 37)	χ^2^ or t	*p*
Age (years)		33.33 ± 3.36	35.84 ± 4.00	34.62 ± 3.87	−2.06 ^a^	0.047 *
Occupation	Yes	16 (88.9)	17 (89.5)	33 (89.2)		1.000 ¶
	No	2 (11.1)	2 (10.5)	4 (10.8)		
Religion	Buddhist	5 (27.8)	5 (26.3)	10 (27.0)		0.447 ¶
	Protestant	2 (11.1)	4 (21.1)	6 (16.2)		
	Catholic	0 (0.0)	2 (10.5)	2 (5.4)		
	None	11 (61.1)	8 (42.1)	19 (51.4)		
Economic status	Below average	1 (5.6)	0 (0.0)	1 (2.7)		1.000 ¶
	Average	14 (77.8)	15 (78.9)	29 (78.4)		
	Good	3 (16.7)	4 (21.1)	7 (18.9)		
Infertility duration (self-reported)	<6 mo	8 (44.4)	6 (31.6)	14 (37.8)		0.773 ¶
	6 mo–<1 y	6 (33.3)	5 (26.3)	11 (29.7)		
	1–<2 y	2 (11.1)	3 (15.8)	5 (13.5)		
	2–<3 y	2 (11.1)	3 (15.8)	5 (13.5)		
	≥3 y	0 (0.0)	2 (10.5)	2 (5.4)		
Has child(ren)	Yes	8 (44.4)	11 (57.9)	19 (51.4)	0.67	0.413
	No	10 (55.6)	8 (42.1)	18 (48.6)		
Previous failed ART	Yes	1 (5.6)	6 (31.6)	7 (18.9)		0.090 ¶
	No	17 (94.4)	13 (68.4)	30 (81.1)		

Note. Values are *n* (%) or M ± SD. ART = assisted reproductive technology. ^a^ Independent-samples *t*-test. ¶ Fisher’s exact test (Fisher–Freeman–Halton test for variables with more than two categories), used when more than 20% of cells had expected counts <5; other categorical comparisons used Pearson’s chi-square test. * *p* < 0.05.

**Table 2:** Baseline comparability of outcome variables (M ± SD).

Outcome Measure	Intervention (*n* = 18)	Comparison (*n* = 19)	t	*p*
Perceived stress (PSS)	22.06 ± 3.96	23.37 ± 5.57	−0.82	0.42
Depressive symptoms (PHQ-9)	7.72 ± 4.38	8.68 ± 2.87	−0.79	0.43
State anxiety (STAI-S)	40.39 ± 5.97	43.11 ± 6.77	−1.29	0.21
Alexithymia (TAS-20)	47.22 ± 9.03	48.53 ± 8.84	−0.44	0.66

Note. PSS = Perceived Stress Scale; PHQ-9 = Patient Health Questionnaire-9; STAI-S = State-Trait Anxiety Inventory–State subscale; TAS-20 = 20-item Toronto Alexithymia Scale. Higher TAS-20 scores indicate greater alexithymia.

**Table 3:** Feasibility outcomes.

Domain	Indicator	Result
Recruitment	Enrolled/screened; recruitment window	40/40 screened and enrolled (20 per condition); June–August 2025; number who saw the notice not recorded (recruitment rate not calculable)
Retention	Completed posttest/enrolled	Overall 37/40 (92.5%); intervention 18/20 (90.0%); comparison 19/20 (95.0%)
Adherence	Sessions completed/prescribed (self-reported)	108/108 (100%); 18/18 completed all six sessions
Engagement	Session duration (self-reported)	Session-level mean duration ranged from 8 to 16 min
Adverse events	Safety incidents; inappropriate model responses; escalations	None reported by participants

**Table 4:** Pretest and posttest descriptive statistics, between-group difference in change, 95% confidence intervals, and effect sizes.

Outcome	Group	Pretest M ± SD	Posttest M ± SD	Within-Group Change	Difference in Change (95% CI)	d
Perceived stress (PSS)	Int.	22.06 ± 3.96	17.78 ± 4.66	−4.28	−5.91 (−9.07, −2.74)	−1.25
	Comp.	23.37 ± 5.57	25.00 ± 4.28	+1.63		
Depressive symptoms (PHQ-9)	Int.	7.72 ± 4.38	7.44 ± 2.73	−0.28	+1.88 (−0.11, +3.87)	+0.63
	Comp.	8.68 ± 2.87	6.53 ± 2.87	−2.16		
State anxiety (STAI-S)	Int.	40.39 ± 5.97	38.06 ± 7.36	−2.33	−3.81 (−7.49, −0.12)	−0.69
	Comp.	43.11 ± 6.77	44.58 ± 7.54	+1.47		
Alexithymia (TAS-20)	Int.	47.22 ± 9.03	42.22 ± 9.50	−5.00	−7.05 (−12.50, −1.61)	−0.86
	Comp.	48.53 ± 8.84	50.58 ± 8.09	+2.05		

Note. Int. = intervention group (*n* = 18); Comp. = comparison group (*n* = 19). Within-group change = posttest − pretest. Difference in change = intervention change − comparison change; negative values favour the intervention group on all four measures, since lower scores indicate less stress, fewer depressive symptoms, lower state anxiety, and less alexithymia. Cohen’s d was computed on the change score using the pooled within-group SD of change. Confidence intervals are not adjusted for multiplicity. Positive values for depressive symptoms indicate that the comparison group improved more than the intervention group.

**Table 5:** Two-way repeated-measures ANOVA for all outcomes.

Outcome	Source	SS	df	MS	F	*p*	ηp^2^
Perceived stress	Time	32.36	1	32.36	2.88	0.098	0.076
	Time × Group	161.39	1	161.39	14.37	<0.001	0.291
	Error (within)	393.02	35	11.23			
	Group	336.67	1	336.67	10.41	0.003	0.229
	Error (between)	1131.46	35	32.33			
Depressive symptoms	Time	27.42	1	27.42	6.15	0.018	0.149
	Time × Group	16.34	1	16.34	3.66	0.064	0.095
	Error (within)	156.07	35	4.46			
	Group	0.01	1	0.01	0.00	0.982	0.000
	Error (between)	592.83	35	16.94			
State anxiety	Time	3.42	1	3.42	0.22	0.639	0.006
	Time × Group	66.98	1	66.98	4.40	0.043	0.112
	Error (within)	533.37	35	15.24			
	Group	394.56	1	394.56	4.86	0.034	0.122
	Error (between)	2844.27	35	81.26			
Alexithymia	Time	40.15	1	40.15	1.21	0.279	0.033
	Time × Group	229.88	1	229.88	6.92	0.013	0.165
	Error (within)	1163.47	35	33.24			
	Group	431.34	1	431.34	3.48	0.071	0.090
	Error (between)	4340.12	35	124.00			

Note. SS = sum of squares; MS = mean square; ηp^2^ = partial eta squared. *p*-values are not adjusted for multiple comparisons across the four outcomes; with a Bonferroni-corrected α of 0.0125, only the interaction and between-subjects effects for perceived stress remain significant.

**Table 6:** Analysis of covariance: posttest scores adjusted for baseline score and prior failed ART experience.

Outcome	Adjusted Mean Difference	95% CI	F (1, 33)	*p*	ηp^2^
Perceived stress (PSS)	−6.69	−9.64, −3.74	21.29	<0.001	0.392
Depressive symptoms (PHQ-9)	+0.82	−0.74, +2.38	1.15	0.292	0.034
State anxiety (STAI-S)	−4.21	−8.23, −0.20	4.56	0.040	0.121
Alexithymia (TAS-20)	−6.85	−12.07, −1.64	7.16	0.012	0.178

Note. Dependent variable: posttest score. Predictors: group, corresponding pretest score, and prior failed ART experience (yes/no).

## Data Availability

The de-identified dataset is available from the corresponding author upon reasonable request.
